# The Elixir of Youth

**Published:** 1921-05-21

**Authors:** 


### The Elixir of Youth.

For many months the English daily newspapers have been giving great prominence to the stories of a new operative treatment for the rejuvenation of the elderly. Various forms of such treatment have been attractively presented through the adroit handling of enthusiastic journalists, but that which held the field until a few days ago was that of the thyroid gland, whose magical properties were supposed to have been discovered by a Viennese professor. Last week, however, an old gentleman named Alfred Wilson, who had recently returned from a visit to the professor, and who had actually hired the Albert Hall in London to inform the public of his marvellous rejuvenation, suddenly died in his bed on the eve of the promised lecture. If the result is to instil a little common-sense into gullible writers and a still more easily gulled public, the experience and death of this deluded old man will not have been in vain. Several leading medical men have seized the occasion to point the moral and to call attention to the danger and folly of these ridiculous claims, most of which appear to originate on the Continent. It is stated by more than one doctor that the frequent and unguarded announcements of the discovery of the elixir of youth have had a disturbing effect on many elderly patients, making them nervously excited, restive, and absurdly confident in their ability to grow young again if only their medical attendants were not so conservative and unimaginative.

The simple fact is, of course, that there is no warrant for any of these alleged "cures"—which are as difficult to authenticate as the miraculous cancer remedies that periodically bring false hopes to thousands of sufferers—and competent persons engaged in medical research are acting in the best

THE PUBLIC WELL-BEING—(*continued*).

interest both of science and the public in emphasising the peril of a procedure which seeks to instil into the human frame an agent whose activities are almost unknown. As one well-known doctor says, until science has completely sifted the changes involved, interference with the natural and physiological cycle of life is bound to be fraught with incalculable dangers. It is perfectly true, as the *Times* points out (in a not very well-timed effort to keep alive the hopes of men in the quest for perpetual youth), that there is no absolute reason why a human life should not be continued at its full power indefinitely, and there is no reason to cast ridicule upon the experiments of those who are engaged upon "the most adventurous research which man has ever conceived." But the prospect of success is so remote and indefinite that to talk of these experiments as rapidly approaching a *fait accompli* is grossly to mis-state the facts. Moreover, even assuming ultimate succes, the achievement would itself raise a tremendous problem that might baffle the wit of man to solve. What is to happen to our already overcrowded populations in a world in which men never grow old? The thought is sufficiently appalling to extinguish finally the gallant efforts of Mr. Harold Cox and all the Malthusiasts to keep a just balance between the fruits of the earth and the number of people who have to share them.

